# Comparison irisin peptide serum level in pregnant women with and without gestational diabetes mellitus: A case–control study

**DOI:** 10.1002/edm2.370

**Published:** 2022-09-18

**Authors:** Zahra Mazloum Khorasani, Majid Besharat, Hassan Mehrad‐Majd, Seyed Ali Mohammadi, Malihe Mahmoudinia

**Affiliations:** ^1^ Associate Professor of Endocrinology, Metabolic Syndrome Research Center Mashhad University of Medical Sciences Mashhad Iran; ^2^ Faculty of Medicine Mashhad University of Medical Sciences Mashhad Iran; ^3^ Molecular Medicine, Cancer Molecular Pathology Research Center Mashhad University of Medical Sciences Mashhad Iran; ^4^ Faculty of Medicine Mashhad University of Medical Sciences (MUMS) Mashhad Iran; ^5^ Assistant Professor of Obstetrics & Gynecology, Fellowship of Infertility, Maternal & Neonatal Research Center, Faculty of Medicines Mashhad University of Medical Sciences Mashhad Iran

**Keywords:** gestational diabetes mellitus, glucose, insulin, irisin, pregnant

## Abstract

**Introduction:**

Gestational diabetes mellitus (GDM) is a metabolic disease that affects mother and foetus during pregnancy, causing acute and chronic adverse effects. Irisin is proposed as a novel marker to predict GDM. The aim of this study was to assess the role of irisin peptide serum levels in gestational diabetes and compare with healthy pregnant women.

**Methods:**

This case–control study was conducted on women at 24 to 34 weeks of gestation in Ghaem Hospital affiliated with Mashhad University of Medical Sciences between May 2016 and June 2019. In two study groups, GDM and non‐GDM women, an association between maternal serum irisin levels and clinical and biochemical parameters were evaluated. Maternal serum irisin levels were measured by an enzyme immunoassay method. Body mass index, serum levels of glucose, oral glucose tolerance test (OGTT), insulin, haemoglobin A1C, homeostatic model assessment of insulin resistance (HOMA IR) and irisin were evaluated.

**Results:**

Totally, 56 participants (30 non‐GDM women and 26 women with GDM) were enrolled. Not statistically significant was observed in serum irisin levels between GDM and non‐GDM women. (*p* = .814) Irisin levels were not significantly associated with maternal age, systolic and diastolic blood pressure, the number of pregnancies, gestational age, fasting blood sugar, insulin, HOMA IR, one‐hour and two‐hour serum glucose and body mass index.

**Conclusions:**

There is no significant difference between GDM and non‐GDM groups in the case of irisin value and later, no association of irisin with metabolic and anthropometric parameters. These findings need to be assessed in future experiments.

## Graphical abstract

Some studies proposed that the serum irisin level can be used as an early biomarker to predict Gestational Diabetes Mellitu (GDM) in pregnancy. This case‐control study was conducted to compare irisin peptide serum levels in GDM and non‐GDM patients. Our finding shows no correlation between serum irisin and the development of GDM.

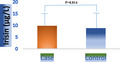

## 1 INTRODUCTION

Gestational diabetes mellitus (GDM) is a common metabolic disease in pregnancy that increased the risk of adverse maternal and foetal outcomes. It is affecting around 13% of pregnant women. The incidence will presumably increase significantly over the next decades because of the percentage of lifestyle problems as obesity becomes more prevalent.^1^

Gestational diabetes mellitus is a form of diabetes diagnosed in the second or third trimester of pregnancy in a woman who has never had diabetes before.^2^

GDM is associated with adverse obstetric complications, such as polyhydramnios, increased caesarean delivery rate and preterm delivery. The foetus suffers from congenital malformation, neonatal respiratory distress syndrome, macrosomia and intrauterine death. It has been suggested that inflammatory markers including tumour necrosis factor‐alpha (TNF‐α), C‐reactive protein (CRP) and interleukin (IL) 6 impact cause insulin resistance and endothelium dysfunction then contribute to the pathogenesis of GDM.^3^

Consequently, early detection of GDM and timely initiation of proper treatment are especially important to reduce adverse complications in pregnancy.^4^ Metabolic syndrome is an increasing global problem and as early detection of these diseases, decreases morbidity and their complications so, researchers are looking to discover new ways to diagnose them. Mutations in some genes, defects in the secretion of placental hormones and destruction of beta cells can all lead to gestational diabetes. In recent years, the role of adipocyte‐derived and hepatocyte‐derived factors in insulin resistance and proinflammatory effects in gestational diabetes has been evaluated.^5^ Also, the role of proteins secreted by myocytes in metabolic diseases has recently received much attention. Myokines are released by myocytes in muscle tissue and are involved in the regulation of metabolism in muscles.^6^

Evidence suggests that myokine dysfunction may play a role in developing insulin resistance, which is a significant cause of gestational diabetes. The myokines such as IL‐6, IL‐13 and FGF21 cause insulin sensitivity.^7^ Irisin is a newly discovered myokine that improves insulin sensitivity.^8^ A novel myokine, Irisin, has been identified substance that drives the brown‐fat‐like conversion of white adipose tissue. Association between serum irisin and various metabolic parameters was evaluated. Several studies have been performed on the effect of irisin on glucose metabolism, which have had contrasting results.^9^, ^10^

Although the results of some studies have shown that people with gestational diabetes have lower circulating irisin levels,^11^, ^12^, ^13^, ^14^ other studies have failed to find a statistically significant difference in serum irisin levels between healthy and GDM pregnant women.^15^, ^16^ Liang Zhao et al.^17^ performed a meta‐analysis on four articles and reported the lower circulating irisin in GDM women compared to others, because there was insufficient information about the link between serum irisin level and gestational diabetes, this study was performed to investigate the association between serum Irisin level and gestational diabetes.

## 2 MATERIALS AND METHODS

This case–control study was performed on 64 pregnant women, including 26 patients with gestational diabetes and 38 healthy pregnant women in Ghaem Hospital affiliated with Mashhad University of Medical Sciences, Mashhad, IRAN between May 2016 and June 2019.

All the patients were informed about the procedure of the study, and informed consent was obtained from them before enrolling in the study. This study was approved by the ethics committee of Mashhad University of Medical Sciences with approval code IR.MUMS.MEDICAL.REC.1397.705.

Pregnant women between the ages of 18–40 years old with 24–34 weeks of pregnancy were included in the study. Exclusion criteria were as follows: history of diabetes and prediabetes, insulin use, and use of anti‐hyperglycaemia drugs and hypertension, and also, patients who were dissatisfied with the continuing study were excluded.

At first, each patient underwent a complete medical and clinical history and obstetrics examination, general examination, including blood pressure (BP), height, and weight as well as body mass index (BMI) recorded. Fasting blood glucose was used to screen at the beginning of pregnancy. If a positive diagnosis is not made, patients are screened at 24‐ to 28‐week gestation with a 2‐h, 75‐g oral glucose tolerance test. According to the American Diabetes Association (ADA) criteria, the diagnosis of gestational diabetes was considered each of the following^18^: A fasting plasma glucose level (FBS) > 92 mg/dl or first‐hour OGTT >180 mg/dl or second‐hour OGTT >153 mg/dl.

Gestational ages were calculated with the last menstrual period and confirmed with sonography from the first trimester. After obtaining informed consent, patients who met the inclusion criteria were introduced to the laboratory to perform tests including fasting blood glucose and glucose tests 1 and 2 h after consuming 75 g of glucose, insulin, HBA1c and irisin.

Five milliliters of blood was taken from the brachial vein in the laboratory. The sample was kept at room temperature for 20 min and then centrifuged (3000 Rpm) for 10 min. Glucose oxidase peroxidase methods have been used to measure blood glucose (Pars Azmoon®, Tehran, Iran). Haemoglobin A1C was determined with the turbidimetry technique (Pars Azmoon®, Tehran, Iran), and also, chemiluminescent immunoassay (CLIA) is used for the measurement of serum insulin concentration (Liaison Insulin kit, Diasorin S.p.A., Saluggia, Italy). Then, samples were stored at minus 20 ° C. Irisin levels were measured using Enzyme‐Linked Immunosorbent Assay (ELISA). (Human irisin Elisa Kit, Shanghaicrystal Day Biotech Co). The minimum detection limit for irisin was 0.05 μg/ml. The intra‐ and inter‐assay coefficient of variation was less than 8% and 10%, respectively. The method developed and previously described by Aydin et al.^19^ was used for the assay validation of the samples.

Insulin resistance was also calculated by the HOMA‐IR method using the following formula: HOMA‐IR = [glucose (nmol/L) * insulin (mU/ml)/22.5], using fasting values.^20^

Finally, patients in both groups GDM and non‐GDM women were compared in terms of metabolic parameters and irisin. The relationship between irisin and each of the parameters in both groups was investigated.

### 2.1 Statistical analysis

Normality was tested with Kolmogorov–Smirnov and histogram diagram, and a t‐test was used to compare quantitative variables between the two groups. Due to the lack of normal distribution of the irisin variable in the histogram diagram, the Mann–Whitney test was used to evaluate the level of irisin for comparison between the two groups. Spearman test was used to evaluate the relationship between the irisin variable and other variables. Data analysis was performed using SPSS 26 software. A *p*‐value of less than 0.05 was considered significant.

## 3 RESULTS

This case–control study was performed on 56 pregnant women, including 26 patients with gestational diabetes and 30 healthy women. The mean age in the healthy pregnant women (control group) was 29.52 ± 6.7, and the mean age in the gestational diabetes group was 32.26 ± 4.7 (*p* = .69).

The mean body mass index in the control group was 26.75 ± 5.05, and the mean body mass index in the group with gestational diabetes was 28.43 ± 5.66 (*p* = .306). The gestational age in the control group was lower than in the gestational diabetes group (*p* = .011).

In total, the two groups were similar in terms of maternal age, the number of pregnancies, body mass index, systolic and diastolic blood pressure. Only the gestational age in the control group was lower than in the gestational diabetes group. Pregnant women with GDM had significantly higher fasting blood glucose FBG (*p* = .004), first‐hour OGTT glucose (*p* = .001), second‐hour OGTT glucose (*p* = .001) as compared to control group. Comparing other variables such as FBS, insulin, HBA1C and HOMA‐IR irisin in both groups are written in the table below. (Table 1).

**TABLE 1:** Demographic and laboratory characteristics of gestational diabetes mellitus (GDM) patients and controls

	Gestational diabetes group *N* = 26	Control group *N* = 26	*p*‐value
Mother's age (years)	32.26 ± 4.7	29.52 ± 6.7	.69
Body mass index (kg/m^2^)	28.43 ± 5.66	26.75 ± 4.87	.306
Gestational age (weeks)	27.5 ± 3.9	24.04 ± 4.15	.011
Systolic blood pressure (mmHg)	109 ± 418.2	104.34 ± 13.34	.176
Diastolic blood pressure (mmHg)	9.42 ± 70	65.35 ± 13.3	.357
Gravida	5 ± 3	1 ± 2	.270
FBS (mg/dl)	93.96 ± 15.42	75.26 ± 8.85	<.0001
OGTT 60	183.16 ± 49.15	125.91 ± 30.71	<.0001
OGTT 120	153.34 ± 44.78	96.71 ± 25.64	<.0001
Insulin(μU/ml)	16.92 ± 15.3	13.33 ± 6.89	.242
Irisin (μg/L)	9.86 ± 10.77	8.86 ± 13.65	.814
HBA1C	4.8 ± 0.46	4.84 ± 0.55	.842
HOMA‐ir	4 ± 3.53	2.53 ± 1.46	.038

Irisin levels were not significantly associated with maternal age, systolic and diastolic blood pressure, the number of pregnancies, gestational age, fasting blood sugar, and one‐hour and two‐hour serum glucose. The relationship between irisin level and demographic and biochemical characteristics is shown in (Table 2).

**TABLE 2:** Correlation between irisin levels and anthropometric and biochemical parameters in patients with gestational diabetes

Variables	*p* value	R correlations
Mother's age (years)	.987	−0.002
Gestational age (weeks)	.338	−0.167
BMI (kg/m^2^)	.9	0.078
SBP (mmHg)	.583	0.095
DBP (mmHg)	.356	0.207
Gravida	.44	−0.125
FBS (mg/dl)	.835	0.028
OGTT1h	.377	−0.124
OGTT2h	.182	−0.186
Insulin(μU/ml)	.467	0.106
HOMA‐IR	.685	0.059

Table 3 shows the relationship of variables with irisin level after removing the effect of gestational age. Although initially body mass index was inversely related to irisin level, after removal of the effect of age pregnancy, there was no correlation between them.

**TABLE 3:** Relationship between irisin level and other variables in control group and gestational diabetes group after removing the effect of gestational age

Variables	*p* value	R correlations
Mother's age (years)	.807	−0.152
BMI (kg/m^2^)	.9	0.078
SBP (mmHg)	.704	0.235
DBP (mmHg)	.888	0.088
Gravida	.429	−0.466
FBS (mg/dl)	.65	0.278
OGTT1h	.698	0.239
OGTT2h	.711	−0.229

## 4 DISCUSSION

In this study, we find out that there was no significant difference between the GDM and non‐GDM women in terms of serum irisin level. Serum irisin level was calculated to be 9.86 ± 10.77 in the gestational diabetes group and 8.86 ± 13.65 in the control group. No correlation was found between irisin level and parameters such as maternal age, gestational age, number of pregnancies, systolic and diastolic blood pressure. Also, irisin level was not significantly associated with FBS, OGTT, insulin, insulin resistance index and BMI.

Irisin is a new myokine, an adipokine that has been shown in animal studies to regulate energy production by converting white adipose tissue to brown, increasing metabolic processes and decreasing insulin resistance. Numerous studies have examined serum irisin levels in the duration of pregnancy in hopes of using irisin levels as a new marker to predict gestational diabetes.

Similar to our study, Ebert et al.^21^ found that there was no significant difference between serum irisin levels in healthy and GDM pregnant women. In their study, there was a direct relationship between serum irisin and fasting insulin levels but we did not detect any correlation between them. Also, Seda et al. confirmed that serum irisin levels were not substantially different in pregnant women with diabetes and there was no association between irisin levels and BMI, HbA1C and maternal age although irisin levels increased in mid‐pregnancy compared to early pregnancy.^16^

Nilufer Celik revealed no significant difference between women with gestational diabetes mellitus and those with a normal glucose tolerance test in relation to the maternal serum irisin. However, it was positively connected with the fasting insulin level, HOMA‐IR and body mass index. They concluded that serum irisin level may control HOMA‐IR value and fasting insulin level in women with GDM.^15^ Contrary to the studies mentioned above, Fadia et al. performed a study on 100 pregnant women and concluded that serum Irisin levels in gestational diabetic women were significantly lower than in healthy pregnant women, but there was no significant association with BMI, serum insulin, and OGTT.^22^ This result was confirmed by some other studies.^23^, ^24^, ^25^

Two studies have recently been conducted. First one by AL‐Ghazali who conducted the study on 60 pregnant women. The mean level of serum irisin in the group with gestational diabetes was considerably lower than the control group,^23^ and Pei Wang et al.^24^ confirmed that irisin serum can be useful as a marker to detect the progress of GDM in high‐risk women in early prevention approaches. Our study also demonstrated no correlation between irisin and other anthropometric and biochemical parametric. Inconsistent with our finding, Piya et al. and Shoukry et al. reported a positive association between irisin, HOMA‐IR and the fasting insulin level in GDM women.^26^, ^27^

Also, Nilufer Celik showed the maternal serum irisin level of the GDM subjects and diastolic blood pressure were in reverse.^15^

As mentioned above, there have been some agreements about the association of Irisin with gestational diabetes in the literature. This discrepancy could be attributed to differences in gestational age, parity, physical activity and even diet. As was mentioned by Nilufer Celik, parity, pregnancy age and the rate of physical activity of the patient are various in most studies.^15^ Daskalopoulou SS identified circulating irisin levels were increased in reply to exercise, and it is elevated with the amount of work that they do.^28^ Besides, Osella et al. recently pointed out that diets rich in vegetable proteins and saturated fatty acids were in positive correlation with serum irisin,^29^ and all these reasons could affect the results.

Another interesting finding from our study is that the amount of irisin and insulin levels in our sample are higher than other studies, but it is nearly similar to the result of Dianatinasab et al.,^30^ who performed a study in another city in Iran. Fedia showed a cut of 2.145 irisin levels, which is very low compared to our study, in Iraghi women helps diagnose diabetic patients. In addition, the proportion of HOMA‐IR in our study is higher than others which hypothesized that race may have an important role on the insulin and irisin levels (23).

The amount of irsin in middle pregnancy was not significant difference in comparison with the early pregnancy but irisin concentration was higher in middle pregnancy compared to the early pregnancy levels.^31^ We evaluated the correlation between irisin level and other variables in control group and gestational diabetes group after removing the effect of gestational age because of discrepancy the gestational age.

The major limitation of this study is conducting a cross‐sectional study limited to a small population. Perhaps, more accurate findings can be made by performing a cohort one in different centres on pregnant women from the beginning of pregnancy. In any case, our study is the first one to evaluate serum irisin levels in women with gestational diabetes in Iran.

## 5 CONCLUSION

In this study, no significant difference was found between serum irisin levels in the group with gestational diabetes and the control group. Also, there was no relationship between serum irisin level and other variables such as FBS, insulin resistance index and BMI.

## AUTHORS CONTRIBUTION

All authors have made contributions to writing the manuscript and interpreting the data, and read and approved the final manuscript.

## FUNDING INFORMATION

Mashhad university of Medical Sciences.

## CONFLICT OF INTEREST

The authors declare there are no conflicts of interest.

## ETHICS STATEMENT

This study was approved by the ethics committee of Mashhad University of Medical Sciences with approval code IR.MUMS.MEDICAL.REC.1397.705, and all participants provided written informed consent before entry the study.

## Data Availability

Data sharing is not applicable to this article as no new data were created or analyzed in this study.
